# Comparing response of buff-tailed bumblebees and red mason bees to application of a thiacloprid-prochloraz mixture under semi-field conditions

**DOI:** 10.1007/s10646-020-02223-2

**Published:** 2020-05-15

**Authors:** Abdulrahim T. Alkassab, Nadine Kunz, Gabriela Bischoff, Jens Pistorius

**Affiliations:** 1 Julius Kuehn-Institut (JKI), Federal Research Centre for Cultivated Plants, Institute for Bee Protection, Messeweg11/12, Braunschweig, Germany; 2 Julius Kuehn-Institut (JKI), Federal Research Centre for Cultivated Plants, Institute for Bee Protection, Königin-Luise-Str. 19, Berlin, Germany

**Keywords:** Bumblebees, Red mason bees, Exposure, Thiacloprid, Prochloraz

## Abstract

Recent studies have reported interspecific differences in how bee species respond to various stressors. Evaluating the exposure and responses of different bee species to plant protection products is considered an essential part of their risk assessment. This study was conducted to assess the impacts of thiacloprid-prochloraz mixture on buff-tailed bumblebees (*Bombus terrestris*) and red mason bees (*Osmia bicornis*) in a *worst-case* scenario under semi-field conditions. Bumblebee colonies or solitary bee trap nests were confined in tunnels with flowering oilseed rape. The recommended maximum application rates of 72 g thiacloprid/ha and 675 g prochloraz/ha were applied as a tank mixture during bee flight in full flowering oilseed rape. Several parameters such as flight and foraging activity, population parameters, and exposure level were investigated. Our results show adverse effects of the combination of thiacloprid and prochloraz on the reproductive performance of red mason bees. The number of cocoons produced by *O. bicornis* was significantly reduced in the treatment compared to the control group. Regarding bumblebees, we found no effects of the thiacloprid-prochloraz mixture on any observed parameters of colony development. The maximum detected concentrations of both active substances three days after application were higher in *O. bicornis* pollen mass compared to *B. terrestris* stored pollen. We conclude that this *worst-case* scenario of thiacloprid-prochloraz exposure poses a high risk to solitary bees and thus the use of such mixture should be restricted.

## Introduction

Plant protection products (PPPs) are continuously assessed for their possible impact on non-target organisms such as honeybees. However, the risk assessment does not always reflect the reality of plant protection use, where several products are mixed in sprayer tanks. The use of PPP mixtures from various classes of chemicals (e.g., insecticides, fungicides, growth regulators, and fertilizers) to control various pests and diseases is common practice in agricultural production systems. Thus, the farmer is able to reduce the production costs and increase the effectivity by controlling a broader spectrum of pests with one application (Das 2014).

However, the interactions of different chemicals in mixtures and their related effects are a great concern to regulatory authorities, e.g. in the United States and Europe (Backhaus et al. 2010).

Bees, due to their foraging activity, are exposed to a wide spectrum of agrochemicals used in bee-attractive crops such as tree fruit orchards and oilseed rape. The mixture exposure to different chemicals has been reported to cause adverse effects within the additive range for the majority of cases (ca. 80%) in environmental toxicology (Belden et al. 2007; Cedergreen 2014). However, several research studies have given special attention to the synergistic effects of mixtures of certain insecticides and fungicides on bees. On honeybees, synergistic negative effects have already been reported for the mixture of ergosterol biosynthesis-inhibiting fungicides (EBI-fungicides) and pyrethroid insecticides, whereas the solo-application of these products is considered to have a low risk (Colin and Belzunces 1992; Pilling and Jepson 1993). Similar synergisms were detected for combinations of EBI-fungicides and some neonicotinoid insecticides under laboratory conditions (Schmuck et al. 2003; Iwasa et al. 2004; Manjon et al. 2018). Recently, the synergy between EBI-fungicides and further insecticide classes, i.e., diamide (Wade et al. 2019) and butenolide (Tosi and Nieh 2019) were also reported in honeybees.

Studies conducted to assess the risk of PPPs were mainly performed with the European honeybee (*Apis mellifera*), which is considered (1) a surrogate species for other bee species due to its high sensitivity and (2) a good environmental indicator of agrochemical pollution.

In a series of screening laboratory experiments on tank mixtures in a spray chamber, synergistic effects on the mortality of adult honeybees were observed for some combinations of pesticides when exposed by contact. The synergisms of products containing the neonicotinoid thiacloprid and different EBI fungicides were confirmed in honeybees (Wernecke et al. 2019). Further higher-tier tests under semi-field and field conditions showed a significant increase of adult mortality of honeybees after application of a tank mixture containing thiacloprid (Biscaya®)-prochloraz (Mirage 45® EC) at recommended application rates during bee flight (Wernecke et al. 2019; Kunz et al. 2017).

A few studies have been conducted to investigate the effects of such mixtures on non-*Apis* bees. Biddinger et al. (2013) reported synergistic effects of fenbuconazole and acetamiprid on the western honeybee *A. mellifera* and the horned-face bee *Osmia cornifrons*. Furthermore, the combination of propiconazole and clothianidin was found to affect *A. mellifera*, *Bombus terrestris* and the solitary bee *Osmia bicornis* (Sgolastra et al. 2017; Robinson et al. 2017; Manjon et al. 2018; Beadle et al. 2019). Raimets et al. (2018) showed that the toxicity of fipronil, cypermethrin, and thiamethoxam to bumblebees is synergized by imazalil. However, most of the reported studies were conducted under laboratory conditions. Therefore, there are requirements to evaluate the observed effects in laboratory by conducting higher tier studies to cover the realistic exposure levels under field conditions.

Though risk assessments of PPPs on honeybees have been extrapolated to non-*Apis* bees (EFSA 2013), several studies have reported interspecific differences between *Apis* and non-*Apis* bee species in responses to various stressors including exposure to agrochemicals (Scott-Dupree et al. 2009; Thompson 2016; Rundlöf et al. 2015; Uhl et al. 2019; Heard et al. 2017; Arena and Sgolastra 2014). It may not always be reasonable to extrapolate from honeybees to evaluate the risk on other bee species, due to the differences in the exposure profiles for each species (Thompson and Hunt 1999). However, up to now, it has remained unknown whether other bee species may be more endangered in the field than honeybees are. Several guidelines are available to conduct these higher-tier studies with honeybees, while there are no guidelines for non-*Apis* bees. Therefore, we conducted the present study to evaluate the risk of a tank mixture containing thiacloprid (Biscaya®) and prochloraz (Mirage 45® EC) on non-*Apis* bees.

Two commercially used non-*Apis* bee pollinators, i.e., *B. terrestris* and *O. bicornis*, were used, which have different life histories. *B. terrestris* is a eusocial species, and each colony consists of a reproductive queen and up to a few hundred of non-reproductive workers, whereas *O. bicornis* is a solitary bee species, in which the female mates with a male after having hatched and is then responsible for the provision of its own brood. To our knowledge, this is the first time that the impacts of tank mixtures on *B. terrestris* and the solitary bee *O. bicornis* as well as their exposure level through different matrices are tested under a *worst-case* scenario (semi-field condition, application of recommended maximum application rate and application during bee flight in full flowering crops).

## Materials and methods

### Test organisms

Bumblebee colonies (*B. terrestris*) were obtained from a commercial supplier (Biobest, Belgium) five days before the start of the experiment. They were delivered in a plastic breeding box surrounded by a cardboard box and kept at room temperature and ambient humidity in a dark room. Each colony consisted of one egg-laying queen and 58.5 ± 12.5 (mean ± SE) worker bees. During the laboratory phase, commercial feeding solution (Biogluc®, Biobest) and pollen were provided ad libitum. Male and female cocoons of *O. bicornis* were ordered from a commercial supplier (WAB-Mauerbienenzucht, Konstanz, Germany) and were placed directly in the tunnels after arrival (Day−12).

### Experimental design

To assess the effect of a tank mixture containing thiacloprid (Biscaya®) and prochloraz (Mirage 45® EC) on performance of buff-tailed bumblebees and red mason bees, several parameters such as flight and foraging activity, population parameters, and exposure level were investigated. The recommended maximum application rate of 72 g thiacloprid/ha and 675 g prochloraz/ha was applied as a tank mixture during bee flight in full flowering oilseed rape (OSR) in a semi-field trial in 2018. The study was conducted at a location located in Lower Saxony, Sickte, Germany. The geographic coordinates were 52°13′22.3″N 10°37′48.1″E. The temperature during the experimental period was 15.87 ± 2.18 °C (for more details see Table S1).

There were two treatment groups; the control C (water) and the test item treatment group T (tank mixture). In total, 27 tunnels were used. The number of replicates was seven tunnels for each control and treatment group in the bumblebees’ setup, and six control tunnels and seven treatment tunnels in the red mason bees’ setup. Each replicate consisted of a tunnel with one colony of bumblebees or trap nest of red mason bees. Each tunnel measured 10 × 4 m and was covered with a net of 1.2 mm mesh width. Two gauze stripes as a ground cover were placed within each tunnel and served for walking and collecting dead bees. The total area of flowering oilseed rape (*Brassica napus*) was ca. 35 m^2^ in each tunnel. To observe foraging activity, three quadrates (1 × 1 m) were established randomly in each tunnel.

Bumblebee colonies were transferred into the tunnels on day 7 before application (DAA -7; day after application (DAA)). The cocoons of red mason bees (40 males and 40 females per tunnel) were placed on day 12 before application (DAA -12). The application of thiacloprid-prochloraz mixture was made on DAA 0 (8^th^ of May 2018) using a backpack sprayer equipped with seven 80-degree TeeJet TP80015 nozzles (nozzle spacing: 250 mm) delivering 200 l/ha. After application, the residual amount in the sprayer was gelatinized back to verify the actual amount applied (deviations ≤ 5%).

The exposure phase lasted 8 days after application (DAA +8). The population assessments were conducted on DAA +3 and DAA +8. At the end of the exposure, bumblebee colonies were closed and transferred into the laboratory. On day 37 after application (DAA +37), the colonies were frozen to assess the development of the colonies. The trap nests were also closed and placed in a protected location until completed cocoon formation to evaluate the number of produced cocoons per trap nest.

### Data collection

#### Flight and foraging activity

The foraging activity, number of forager bees on the crop, was assessed visually for one minute in three quadrates of the flowering oilseed rape (OSR). Furthermore, the flight activity, number of returning bees at the entrance of the nest/colony, was counted visually for 1 min. These assessments were conducted daily during the experimental period from DAA −3 to DAA +7. On the day of application the activity was recorded twice immediately before (DAA −0) and 2 h after application (DAA +0).

#### Population parameters

##### Osmia bicornis

The number of cells built in the nesting cavities was counted by taking a picture on the assessment dates (DAA −1, +3, and +8). The number of cocoons produced per trap nest was counted in autumn. The termination rate was calculated as the difference between the number of brood cells on DAA +8 and the number of produced cocoons. The sex ratio was determined by sorting the cocoons by weight and size.

##### Bombus terrestris

The number of worker bees and the number of brood cells (i.e. eggs, larvae, and pupae cells) were evaluated before placement in the tunnel by taking a picture of each colony in the laboratory under red-light conditions. After placement in the tunnel, two population estimations were conducted by taking a picture and weighing each colony on DAA +3 and DAA +8. Additionally, samples of stored pollen and nectar were collected from the colony. Colony weight and the number of dead bees inside the colonies were recorded on each assessment day. During the development phase in the laboratory, after the exposure phase, two further population estimations were conducted on DAA +19 and DAA +37. The counting of brood cells on the images was performed by using ImageJ software.

#### Residue analysis

Samples of different matrices, i.e. OSR-flowers, stored pollen and nectar from the bumblebee colony; pollen mass and mud walls from the trap nests of red mason bees were collected at different assessment days to evaluate the exposure level over the experimental period. To evaluate the residue concentration at different plant horizons, filter papers (25 mm, Whatman®) were fixed between plants, with three filters fixed on the flower horizon (*n* = 7 tunnels for each *O. bicornis* and *B. terrestris*) in addition to three filters on a level 30 cm below (*n* = 3 tunnels for each species). All samples were stored at −20 °C.

For residue analysis nectar, pollen and pollen mass samples were weighed in a glass centrifuge tube and a surrogate standard solution (prochloraz-d4 and thiacloprid-d4) and a defined amount of an acetone/water-mixture was added. The tubes were closed and left at room temperature for 30 min. The samples were homogenized with a disperser (MICCRA) and subsequently centrifuged. For the homogenization of the plant samples a waring blender was used because of the bigger sample amounts. An aliquot of the homogeneous mixture was taken for centrifugation. After centrifugation, a defined amount of the supernatant was removed mixed with sodium chloride-solution and transferred onto ChemElut® cartridges. After incubation for 15 min, the samples were eluted with dichloromethane. The eluates were evaporated to dryness and the residual extract was re-dissolved with acetonitrile containing the isotopically labeled internal standard (tebuconazole-d6) using an ultrasonic device. The filtered sample extracts were analyzed by liquid chromatography mass spectrometry (LC-MS/MS). The system used was a Nexera X2 HPLC (SHIMADZU) coupled to a triple quadrupole mass spectrometer Q TRAP 6500+ (SCIEX) equipped with an electro spray ionization (ESI) source. The quantification was carried out by the internal standard method using matrix-matched calibration standards. After dilution (at least 1:100), sample extracts were quantified with standards in solvent.

For residue analysis of filter papers and mud walls, samples were placed in suitable vessels, the surrogate standard solution (prochloraz-d4 and thiacloprid-d4) and a defined amount of acetone were added. After incubation for 30 min, samples were extracted by using a shaking device and subsequently an ultrasonic device (10 min each). Mud wall samples were filtered and an aliquot of the extracts evaporated to dryness, re-dissolved and analyzed as described above. In case of the filters, the combined extracts were evaporated to dryness, re-dissolved and analyzed as described above. The limit of quantification (LOQ) and limit of detection (LOD) are reported in Table 1.

**Table 1:** Limit of quantitation (LOQ), limit of detection (LOD) and relative recovery (REC) for different matrices

Organism	Matrices	Thiacloprid			Prochloraz		
		REC %	LOD µg/kg	LOQ µg/kg	REC %	LOD µg/kg	LOQ µg/kg
*B. terrestris*	Stored pollen from colony	83	0.67	1.33	85	1.33	2.67
	Nectar from colony	105	0.07	0.33	85	0.67	1.33
*O. bicornis*	Mud walls from nest	89	0.13	0.27	82	0.27	0.53
	Pollen mass from nest	83	0.13	0.27	85	0.27	0.67
*B. napus*	Flower	98	0.04	0.09	119	0.18	0.44

### Statistical analysis

The count data of the number of foraging bees and the flight activity at the entrance of red mason bees and bumblebees were analyzed using generalized linear mixed models (GLMM) and Poisson or Gaussian distributions were used where appropriate. Assumptions of Gaussian distribution and homogeneity of variance were met for all other data, i.e., number of bees or cells per colony/nest. The differences between treatments were analyzed using generalized linear mixed models (GLMM) with “treatment” as fixed factor and “replicate” as random factor. Furthermore, the Mann–Whitney U test and Kruskal–Wallis test were used to examine the differences between residues on the filter paper at different levels and residues in different matrices, respectively. All data analyses were conducted using SPSS v. 25 (SPSS Inc., Chicago, IL, USA) at the significance level of 0.05. Summary of statistical results is provided as supplementary information (Tables S2–S6).

## Results

### Flight and foraging activity

**Fig. 1:**
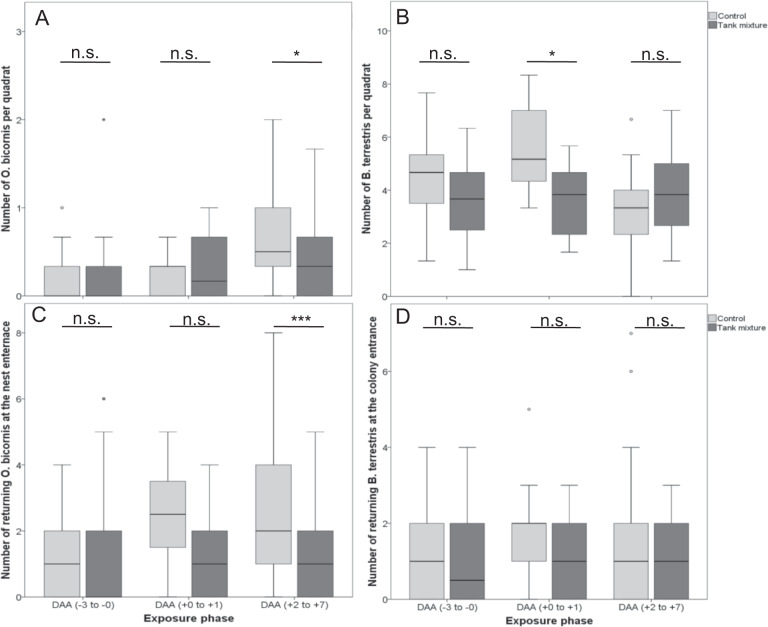
**a** Number of *O. bicornis* individuals per quadrate. **b** Number of *B. terrestris* individuals per quadrate. **c** Number of returning *O. bicornis* female at the nest trap entrance. **d** Number of returning *B. terrestris* individuals at the colony entrance over the exposure phases expressed as day after application (DAA). Treatments are shown as boxplots with median; the edges of the box indicate the 25^th^ and 75^th^ percentiles. Outliers are shown as circles. Asterisks indicate significant differences (*p < 0.05, ***p < 0.001); n.s. indicates non-significant differences

No significant differences were found in the foraging activity or flight activity at the nest entrance of red mason bees between the control and treatment groups, neither pre- nor immediately post-application of the tank mixture (GLMM, *p* > 0.05; Fig. 1a). The foraging activity as well as flight activity at the nest entrance showed significant reduction in the treatment group compared to the control group after application (GLMM, *p* < 0.05; Fig. 1a and c). Generally, the observed activity of red mason bees was low in both variants due to short observation duration of one minute.

Regarding bumblebees, there were no significant differences on the foraging activity between the control and treatment groups neither pre- nor post-application of the tank mixture (GLMM, *p* > 0.05; Fig. 1b). Though a significant reduction of foraging activity was observed immediately after application, the effect was transient (GLMM, *p* < 0.05; Fig. 1b). The flight activity at the entrance of bumblebee colonies did not differ significantly between the control and treatment group over the exposure phase (GLMM, *p* > 0.05; Fig. 1d).

### Population parameters

**Fig. 2:**
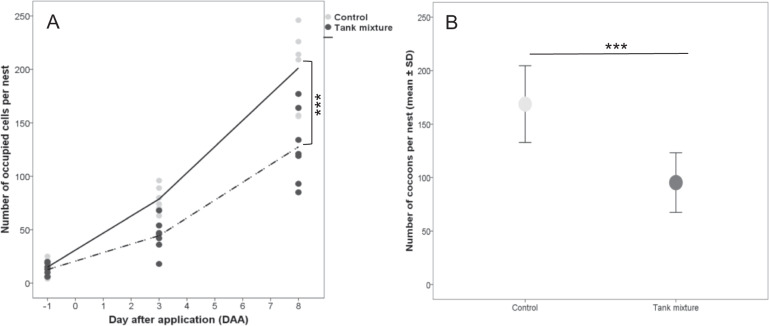
**a** Number of occupied cells per *O. bicornis* trap nest in relation to days after application. **b** Number of cocoons per *O. bicornis* trap nest at the end of experiment (mean ± SD). Asterisks indicate the significant differences (***p < 0.001); n.s. indicates non-significant differences, *n* = 7 for treatment and 6 for control

Adverse effects for the combination of thiacloprid and prochloraz were observed on *O. bicornis*. The number of occupied cells and the number of produced cocoons was significantly reduced (GLMM, *p* < 0.05; Fig. 2a, b). The reduction in produced cocoons was 43.5% compared to control. Otherwise, no effects of the tested combination on the sex ratio per unit were observed, where the percentage of female cocoons produced per nest was 25.5 ± 5.4 and 25.7 ± 10.5% (mean ± SD; GLMM, p > 0.05) in control and treatment group, respectively. The brood termination rate per nest (non-development from cells with eggs on DAA + 8 to cocoons) was low in both variants (control: 3.8 ± 1.9%, treatment: 4.7 ± 2.9, mean ± SD; GLMM, *p* > 0.05).

**Fig. 3:**
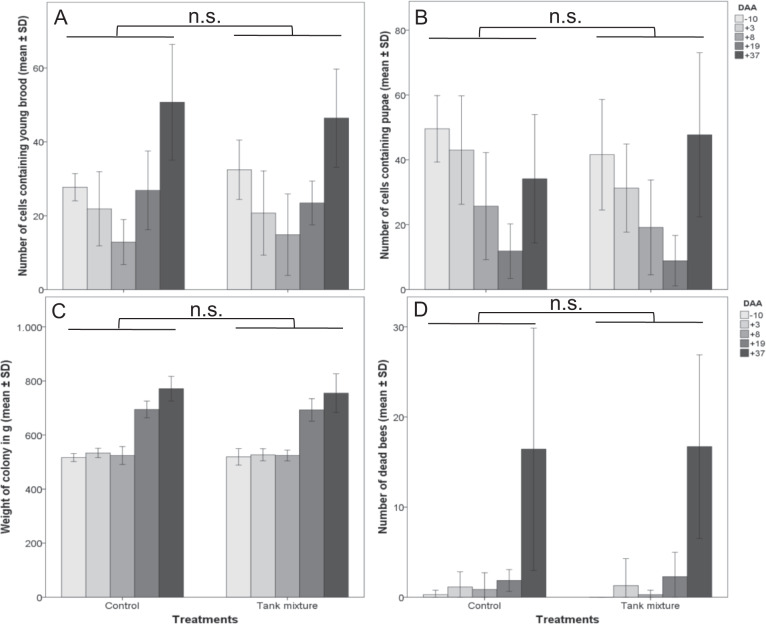
Different parameters of development of *B. terrestris* colonies over the experimental period. **a** Number of eggs and larval cells. **b** Number of pupae cells. **c** Weight of colony. **d** Number of dead workers (mean ± SD). N.s. indicates non-significant differences; *n* = 7. Comparisons of different parameters between treatment and control were conducted at each individual assessment day

No effects of the tested combination on all observed parameters of *B. terrestris* colony development could be found (GLMM, *p* > 0.05; Fig. 3a–d). During the exposure phase in the tunnels, the number of dead bees inside the treated colonies was low and did not differ significantly compared to the control group. Over the experimental period, colonies grew larger and more dead bees accumulated inside the colonies in a similar magnitude for both the treatment and control groups (Fig. 3). Colonies of both variants contained fewer brood cells (larvae and pupae) during the exposure period compared to initial brood assessment but gained brood cells during the post-exposure period. At the end of the experiment, there were no differences between control and treatment colonies in the number of workers (control: 225.14 ± 44.59; treatment: 260.29 ± 31.55 (mean ± SD; GLMM, *p* > 0.05)) the number of males (control: 9.57 ± 18.70; treatment: 2.57 ± 6.37 (mean ± SD; GLMM, *p* > 0.05)).

### Exposure level

**Fig. 4:**
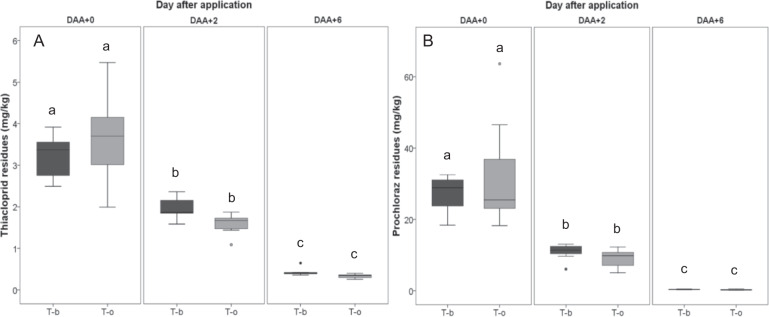
Residue concentration in OSR-flowers at different dates relative to application. **a** Thiacloprid residues. **b** Prochloraz residues. T-b: Treatment *B. terrestris*; T-o: Treatment *O. bicornis*. Treatments are shown as boxplots with median; the edges of the box indicate the 25^th^ and 75^th^ percentiles. Outliers are shown as circles. Boxplots sharing the same letter do not differ significantly at *p* < 0.05; *n* = 7

The maximum concentrations of both active substances were detected in the collected OSR-flowers on the application day (Fig. 4). The residues of prochloraz were 7–9-folds higher than thiacloprid depending on the application rate of 72 g thiacloprid/ha and 675 g prochloraz/ha. The residue concentrations declined significantly over the experimental period (Kruskal–Wallis test, *p* < 0.05). On average, 11.3% of thiacloprid and 1.2% of prochloraz were detected in flowers 6 days after application (Fig. 4). No residues of thiacloprid and prochloraz were detected in the OSR-flowers before application in all tunnels as well as after application in all control tunnels.

Our results showed that residue concentrations 30 cm below flower horizon (1.46 µg thiacloprid/filter; 11.07 µg prochloraz/filter (median values)) were ~33% significantly lower than at the flower horizon (2.01 µg thiacloprid/filter; 18.1 µg prochloraz/filter (median values; Mann and Whitney U test, *p* < 0.05)). This in turn indicates low exposure likelihood on side shoots compared to the main shoots.

**Fig. 5:**
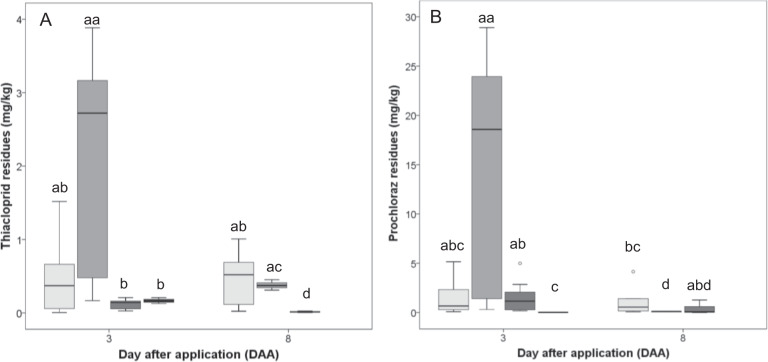
Residue concentration in different matrices (pollen, nectar, mud walls), at DAA +3 and DAA +8, from *B. terrestris* colonies or *O. bicornis* trap nests. **a** Thiacloprid residues. **b** Prochloraz residues. Treatments are shown as boxplots with median; the edges of the box indicate the 25^th^ and 75^th^ percentiles. Outliers are shown as circles. Boxplots sharing the same letter do not differ significantly at *p* < 0.05; *n* = 6 for pollen from the *B. terrestris* colonies, *n* = 3 for nectar from the bumblebee colonies, *n* = 7 for pollen or mud walls from the *Osmia O. bicornis* trap nests

There were no significant differences between residues in *O. bicornis* pollen mass compared to *B. terrestris* stored pollen (Kruskal–Wallis test, *p* > 0.05; Fig. 5) which might have been affected by a high variability in the *O. bicornis* samples. However, the maximum detected concentrations of both active substances on DAA +3 were higher in the *O. bicornis* pollen mass compared to *B. terrestris* stored pollen. The maximum detected concentrations in *O. bicornis* pollen mass were 3.88 mg/kg for thiacloprid and 28.91 mg/kg for prochloraz, whereas in *B. terrestris* stored pollen maxima of 1.52 mg/kg for thiacloprid and 5.51 mg/kg for prochloraz were detected (Fig. 5). On DAA +8, only residues of prochloraz were significantly declined in *O. bicornis* pollen mass (Kruskal–Wallis test, *p* > 0.05; Fig. 5), whereas no declining of both active substances in *B. terrestris* stored pollen was found. Detected residues of both active substances were significantly lower in *O. bicornis* mud walls compared to pollen (Kruskal–Wallis test, p > 0.05). The maximum detected residues in *B. terrestris* nectar on day +3 was 0.21 and 0.03 mg/kg for thiacloprid and prochloraz, respectively (Fig. 5).

## Discussion

Our aim was (1) investigate the response of non-*Apis* bees to a tank-mixture of the neonicotinoid insecticide thiacloprid and the fungicide prochloraz, which were already shown to cause a detrimental synergistic interaction in honeybees (Wernecke et al. 2019) (2) to evaluate the differences in exposure level and related intake amount of non-*Apis* bee species. Generally, eusocial bees which build colonies and are considered as superorganism might be less sensitive against different stressors due to the buffering capacity of the colony (Straub et al. 2015). Nevertheless, the effects on each individual of solitary bee species are expected to have a direct impact on reproductive success (Sandrock et al. 2014).

Our study shows that the tested combination negatively affects the reproductive performance, i.e. number of produced cocoons, of the solitary bee *O. bicornis*. The effect size of the treatment was 43.5% reduction in the number of produced cocoons per nest compared to the control. This effect can be expected to correlate with negative dynamics and fitness losses of *O. bicornis* populations in agricultural environments. Thus, exposure to a tank mixture containing thiacloprid and prochloraz causes a high risk for solitary bees. Nevertheless, care must be taken in extrapolation from semi-field results to global consequences for species population under natural conditions.

Furthermore, our results showed that the flight activity at the nest entrance was significantly reduced after application in the treated group compared to control. This observation might be explained by the effects on the detoxification process after exposure to P450 inhibitors such as EBI-fungicides, which increase in turn the sensitivity of bees to thiacloprid (Beadle et al. 2019; Manjon et al. 2018). A reduced flight activity can be related to various factors such as reducing the survival time of exposed females or effects on nest recognition and the number of foraging trips. However, Beadle et al. (2019) showed no induction in P450 enzymes of *O. bicornis* after being exposed to sublethal concentrations of thiacloprid alone; which might indicates a constitutive expression of P450 enzymes providing protection against this insecticide in single application. Moreover, the insecticide toxicity on honeybees was found to be dependent on the fungicide dose (Thompson et al. 2014).

The detected residues on the application day on the filter paper with an area of 4.91 cm^2^ showed that approximately 0.4 µg thiacloprid and 4.0 µg of prochloraz were deposited on each cm^2^. This corresponds to the mixture ratio of 1:9.3 (72 g a.i. thiacloprid and 675 g a.i. prochloraz). Poquet et al. (2014) reported that the topical exposure surface area of honey bees is ~1.11 cm^2^. Due to non-available information regarding solitary bees and the relative similarity in the body size of *A. mellifera* and *O. bicornis*, we assume that each bee can receive 0.44 µg thiacloprid and 4.4 µg of prochloraz when they forager during the spray application.

Contrary to a reduction in flight activity, our results indicate a low termination rate per nest in both variants, with more than 95% of larvae developing into cocoons; even though relatively high residue concentrations (up to 3.88 mg/kg for thiacloprid and 28.91 mg/kg for prochloraz) were detected in pollen during the first three days after application. This could be related to the high level of antioxidants and thus the detoxification ability during the larval stage of *O. bicornis* (Dmochowska-Ślęzak et al. 2015). Another study suggested that the effects of neonicotinoid exposure may be less severe for *O. bicornis* larvae than for adults (Nicholls et al. 2017). Our results showed male-biased offspring sex ratios in both variants under semi-field conditions. This shift in offspring sex allocation strategies is reported to be related to a poor-quality environment under semi-field conditions (Seidelmann et al. 2010; Strobl et al. 2019).

In this study, bumblebees (*B. terrestris*) seem to be less sensitive to the applied tank mixture in comparison to solitary bees since no significant differences were found between treated and untreated colonies in the various measured response variables including flight activity and colony development. The residues detected in *B. terrestris* stored pollen seem to be lower compared to *O. bicornis* pollen mass, however with high variability between the *O. bicornis* samples. This may be caused by the sampling method, where pollen from day 0 to day 3 was pooled for analysis from different cells inside one trap nest. The variability in *B. terrestris* stored pollen was lower, perhaps because of bumblebees do not have large pollen storages but used the limited pollen inside the tunnels directly for feeding. Raimets et al. (2018) did not find synergism between EBI-fungicide (imazalil) and neonicotinoids (imdiacloprid and thiamethoxam) after dietary exposure in bumblebees. The available laboratory comparative studies indicated in most cases that bumblebees are less sensitive than solitary bee species (Robinson et al. 2017; Sgolastra et al. 2017).

However, none of the bumblebee colonies were producing new queens over the laboratory phase (28 days) after exposure. To characterize the effects of exposure on colony fitness and due to lack of standard methodologies to assess pesticide effects on bumblebee colonies for semi-field trials, we kept colonies in the laboratory after the exposure phase to eliminate the possibility of ongoing uncontrolled exposure in the landscape when the colony was moved and placed at a monitoring site. Although the colonies grew well in our study, containing more than 250 individuals at the end of the experimental periods, it seems that they did not achieve the threshold to start with gyne production. Some studies indicated that only very large bumblebee colonies could produce gynes (Müller and Schmid-Hempel 1992). Nevertheless, several semi-field and field studies reported a lack of queen productions in the tested colonies regardless of the treatments (Siviter et al. 2018; Bernauer et al. 2015). Hence, further optimization steps are still required, where the failure or success thresholds for queen production need to be determined (Cabrera et al. 2016).

Finally, we could show that the testing design, i.e. semi-field tests, can be considered appropriate to investigate the impact of PPPs on solitary bees. However, our results demonstrate the necessity to extend the observation duration of flight activity at the entrance for more than one min in the case of solitary bees and bumblebees, due to low individual numbers. Thus, a prolonged observation duration of up to 3 min could optimize the output of this parameter. Further optimization steps should be taken into account regarding semi-field tests, because of the absence of reliable bumblebee colony development with both the control and treatment groups lacking new queens. This may be due to the limited food resources in the tunnels, where the use of smaller colonies, with maximum one bee per m2 and an extended foraging phase in the tunnel of 3–4 weeks, could improve the test methods.

In conclusion, a high risk is found in this *worst-case* scenario of the mixture of thiacloprid and prochloraz for solitary bees. Both for this reason and because of the previously determined high risk on honeybees under field conditions (Kunz et al. 2017), the application of such mixtures on flowering plants is now restricted in Germany (Federal Office of Consumer Protection and Food Safety 2018).

## Supplementary information

Supplementary Tables
