# Supplementary material for: Comparing response of buff-tailed bumblebees and red mason bees to application of a thiacloprid-prochloraz mixture under semi-field conditions
Source: Ecotoxicology. 2020 May 15;29(7):846–55. doi: 10.1007/s10646-020-02223-2 (PMC7427708; doi:10.1007/s10646-020-02223-2)
Supplement: Supplementary file 1 — Supplementary Tables [file 10646_2020_2223_MOESM1_ESM.docx]

Table S1: Weather data of the study location during the experimental period.

| **DAA** | **Date** | **Air temperature**  **°C**  **[MEAN]** | **Relative humidity**  **%**  **[MEAN]** | **Precipitation**  **mm**  **[MEAN]** |
| --- | --- | --- | --- | --- |
| -3 | 05.05.2018 | 11.89 | 58.81 | 0 |
| -2 | 06.05.2018 | 14.06 | 65.95 | 0 |
| -1 | 07.05.2018 | 15.81 | 62.95 | 0 |
| 0 | 08.05.2018 | 17.13 | 60.61 | 0 |
| 1 | 09.05.2018 | 17.92 | 66.72 | 0 |
| 2 | 10.05.2018 | 16.25 | 82.49 | 0.18 |
| 3 | 11.05.2018 | 13.92 | 78.44 | 0 |
| 4 | 12.05.2018 | 16.45 | 81.02 | 0 |
| 5 | 13.05.2018 | 19.33 | 69.27 | 0 |
| 6 | 14.05.2018 | 18.35 | 54.63 | 0.04 |
| 7 | 15.05.2018 | 15.72 | 64.93 | 0.33 |

| **Endpoints** | **Effect measure** | **F/t** | ***P*-value** |
| --- | --- | --- | --- |
| **Number *O. bicornis* per quadrat** | Treatment | 1.560 | 0.216 |
|  | Phase | 4.360 | **0.016** |
|  | Treatment * Phase | 3.009 | 0.056 |
|  | Treatment * DAA (-3 to -0) | -0.038 | 0.692 |
|  | Treatment * DAA (+0 to +1) | -0.125 | 0.321 |
|  | Treatment * DAA (+2 to +7) | 0.511 | **0.030** |
| **Number of *O. bicornis* at the nest entrance** | Treatment | 7.445 | **0.007** |
|  | Phase | 7.195 | **0.001** |
|  | Treatment * Phase | 5.777 | **0.004** |
|  | Treatment * DAA (-3 to -0) | -0.264 | 0.371 |
|  | Treatment * DAA (+0 to +1) | 1.083 | 0.052 |
|  | Treatment * DAA (+2 to +7) | 1.101 | **0.001** |
| **Number *B. terrestris* per quadrat** | Treatment | 8.331 | **0.004** |
|  | Phase | 6.866 | **0.001** |
|  | Treatment * Phase | 9.607 | < **0.0001** |
|  | Treatment * DAA (-3 to -0) | 0.631 | 0.096 |
|  | Treatment * DAA (+0 to +1) | 2.024 | < **0.0001** |
|  | Treatment * DAA (+2 to +7) | -0.571 | 0.065 |
| **Number of *B. terrestris* at the colony entrance** | Treatment | 4.776 | **0.030** |
|  | Phase | 0.807 | 0.448 |
|  | Treatment * Phase | 0.636 | 0.531 |
|  | Treatment * DAA (-3 to -0) | 0.393 | 0.174 |
|  | Treatment * DAA (+0 to +1) | 0.714 | 0.116 |
|  | Treatment * DAA (+2 to +7) | 0.167 | 0.469 |

Table S2: Result of the statistical analysis of flight and foraging activity of the tested bee species under semi-field conditions over different exposure phases according to generalized linear mixed-effects model (GLMM)

Table S3: Result of the statistical analysis of different parameters of development of *B. terrestris* colonies over the experimental period according to generalized linear mixed-effects model (GLMM).

| **Endpoints** | **Effect measure** | **F/t** | **P-value** |
| --- | --- | --- | --- |
| **Number of cells containing young brood** | Treatment | 0.003 | 0.960 |
|  | DAA | 20.742 | < **0.0001** |
|  | Treatment * DAA | 0.442 | 0.778 |
|  | Treatment * DAA -10 | -0.865 | 0.390 |
|  | Treatment * DAA +3 | 0.208 | 0.836 |
|  | Treatment * DAA +8 | -0.380 | 0.705 |
|  | Treatment * DAA +19 | 0.630 | 0.531 |
|  | Treatment * DAA +37 | 0.787 | 0.435 |
| **Number of cells containing pupae** | Treatment | 1.322 | 0.255 |
|  | DAA | 10.770 | < **0.0001** |
|  | Treatment * DAA | 0.173 | 0.952 |
|  | Treatment * DAA -10 | 1.127 | 0.264 |
|  | Treatment * DAA +3 | 1.650 | 0.104 |
|  | Treatment * DAA +8 | 0.952 | 0.358 |
|  | Treatment * DAA +19 | 0.422 | 0.674 |
|  | Treatment * DAA +37 | -0.040 | 0.968 |
| **Number of dead bees** | Treatment | 0.041 | 0.841 |
|  | DAA | 59.736 | < **0.0001** |
|  | Treatment * DAA | 0.885 | 0.479 |
|  | Treatment * DAA -10 | 1.832 | 0.073 |
|  | Treatment * DAA +3 | -0.200 | 0.842 |
|  | Treatment * DAA +8 | 1.707 | 0.094 |
|  | Treatment * DAA +19 | -0.637 | 0.527 |
|  | Treatment * DAA +37 | 2.173 | 0.899 |
| **Weight of colony in g** | Treatment | 0.264 | 0.609 |
|  | DAA | 138.135 | < **0.0001** |
|  | Treatment * DAA | 0.153 | 0.961 |
|  | Treatment * DAA -10 | -0.132 | 0.896 |
|  | Treatment * DAA +3 | 0.344 | 0.732 |
|  | Treatment * DAA +8 | -0.007 | 0.994 |
|  | Treatment * DAA +19 | 0.088 | 0.930 |
|  | Treatment * DAA +37 | 0.856 | 0.395 |

Table S4: Result of the statistical analysis of number of occupied cells per *O.* *bicornis* trap nest related to the days after application of the tank mixture as well as number of cocoons per *O.* *bicornis* trap nest at the end of experiment according to generalized linear mixed-effects model (GLMM).

| **Endpoints** | **Effect measure** | **F/t** | **P-value** |
| --- | --- | --- | --- |
| **Number of occupied cells per nest** | Treatment | 36.160 | < **0.0001** |
|  | DAA | 209.055 | < **0.0001** |
|  | Treatment * DAA | 11.311 | < **0.0001** |
|  | Treatment * DAA -1 | 0.300 | 0.766 |
|  | Treatment * DAA +3 | 3.281 | **0.002** |
|  | Treatment * DAA +8 | 6.970 | < **0.0001** |
| **Number of cocoons per nest** | Treatment | 35.618 | < **0.0001** |

Table S5: Result of the statistical analysis of residue concentration in OSR-flowers related to the days after application of the at tank mixture according to Kruskal-Wallis test.

| **Endpoints** | **Phase** | **Effect measure** | ***H-test statistic*** | ***P*-value** |
| --- | --- | --- | --- | --- |
| **Tiacloprid residues** | DAA + 0 vs. DAA +2 | Residue concentration | 13.714 | **0.009** |
|  | DAA + 0 vs. DAA +6 | Residue concentration | 27.857 | < **0.0001** |
|  | DAA + 2 vs. DAA +6 | Residue concentration | 14.143 | **0.007** |
| **Prochloraz residues** | DAA + 0 vs. DAA +2 | Residue concentration | 14.000 | **0.008** |
|  | DAA + 0 vs. DAA +6 | Residue concentration | 28.000 | < **0.0001** |
|  | DAA + 2 vs. DAA +6 | Residue concentration | 14.000 | **0.008** |

Table S6: Result of the statistical analysis of residue concentration in different matrices related to the days after application of the at tank mixture according to Kruskal-Wallis test.

| **Endpoints** | **Phase** | **Effect measure** | ***H-test statistic*** | ***P*-value** |
| --- | --- | --- | --- | --- |
| **Thiacloprid residues** | DAA + 3 | P-B3 vs. P-O3 | 0.737 | 0.391 |
|  |  | P-B3 vs. M-O3 | 0.066 | 0.797 |
|  |  | P-B3 vs. N-B3 | 0.225 | 0.635 |
|  |  | P-O3 vs. N-B3 | 4.286 | **0.038** |
|  |  | P-O3 vs. M-O3 | 7.143 | **0.008** |
|  |  | M-O3 vs. N-B3 | 0.476 | 0.490 |
|  | DAA +8 | P-B8 vs. P-O8 | 1.887 | 0.170 |
|  |  | P-B8 vs. M-O8 | 6.199 | **0.013** |
|  |  | P-O8 vs. M-O8 | 14.000 | 0.000 |
|  | DAA +3 * DAA+8 | P-B3 vs. P-B8 | 0.000 | 1.000 |
|  |  | P-B3 vs.P-O8 | 0.066 | 0.797 |
|  |  | P-B3 vs. M-O8 | 6.198 | **0.013** |
|  |  | P-O3 vs. P-O8 | 2.571 | 0.109 |
|  |  | P-O3 vs. M-O8 | 14.000 | < **0.0001** |
|  |  | N-B3 vs. P-B8 | 3.600 | 0.058 |
|  |  | N-B3 vs. P-O8 | 4.286 | **0.038** |
|  |  | N-B3 vs. M-O8 | 4.286 | **0.038** |
|  |  | M-O3 vs P-B8 | 1.887 | 0.170 |
|  |  | M-O3 vs. P-O8 | 14.000 | < **0.0001** |
|  |  | M-O3 vs. M-O8 | 14.000 | < **0.0001** |
| **Prochloraz residues** | DAA + 3 | P-B3 vs. P-O3 | 0.737 | 0.391 |
|  |  | P-B3 vs. M-O3 | 0.737 | 0.391 |
|  |  | P-B3 vs. N-B3 | 3.000 | 0.058 |
|  |  | P-O3 vs. N-B3 | 4.286 | **0.038** |
|  |  | P-O3 vs. M-O3 | 2.571 | 0.109 |
|  |  | M-O3 vs. N-B3 | 4.286 | **0.038** |
|  | DAA +8 | P-B8 vs. P-O8 | 6.198 | **0.013** |
|  |  | P-B8 vs. M-O8 | 1.887 | 0.170 |
|  |  | P-O8 vs. M-O8 | 0.286 | 0.593 |
|  | DAA +3 * DAA+8 | P-B3 vs. P-B8 | 1.333 | 0.248 |
|  |  | P-B3 vs.P-O8 | 6.198 | **0.013** |
|  |  | P-B3 vs. M-O8 | 1.887 | 0.170 |
|  |  | P-O3 vs. P-O8 | 14.000 | < **0.0001** |
|  |  | P-O3 vs. M-O8 | 2.571 | 0.109 |
|  |  | N-B3 vs. P-B8 | 3.600 | 0.058 |
|  |  | N-B3 vs. P-O8 | 4.286 | **0.038** |
|  |  | N-B3 vs. M-O8 | 4.286 | **0.038** |
|  |  | M-O3 vs P-B8 | 0.737 | 0.391 |
|  |  | M-O3 vs. P-O8 | 14.000 | < **0.0001** |
|  |  | M-O3 vs. M-O8 | 2.571 | 0.109 |
